# Bridging human chaperonopathies and microbial chaperonins

**DOI:** 10.1038/s42003-019-0318-5

**Published:** 2019-03-15

**Authors:** Everly Conway de Macario, Masafumi Yohda, Alberto J. L. Macario, Frank T. Robb

**Affiliations:** 1 Department of Microbiology and Immunology, School of Medicine, University of Maryland at Baltimore-Institute of Marine and Environmental Technology (IMET), Columbus Center, Baltimore, MD USA; 2 Department of Biotechnology and Life Science, Tokyo University of Agriculture and Technology, Koganei, Tokyo Japan; 3 Institute of Global Innovation Research, Tokyo University of Agriculture and Technology, Koganei, Tokyo Japan; 4 Euro-Mediterranean Institute of Science and Technology (IEMEST), Palermo, Italy; 5 Institute for Bioscience and Biotechnology Research (IBBR), Rockville, MD USA

## Abstract

Chaperonins are molecular chaperones that play critical physiological roles, but they can be pathogenic. Malfunctional chaperonins cause chaperonopathies of great interest within various medical specialties. Although the clinical-genetic aspects of many chaperonopathies are known, the molecular mechanisms causing chaperonin failure and tissue lesions are poorly understood. Progress is necessary to improve treatment, and experimental models that mimic the human situation provide a promising solution. We present two models: one prokaryotic (the archaeon *Pyrococcus furiosus*) with eukaryotic-like chaperonins and one eukaryotic (*Chaetomium thermophilum*), both convenient for isolation-study of chaperonins, and report illustrative results pertaining to a pathogenic mutation of CCT5.

## Abstract (web summary)

Everly Conway de Macario et al. present a review of microbial models of human chaperonopathies. They discuss the recent progress in using microbes to model human pathogenic mutations and to elucidate disease mechanisms.

## Introduction

The chaperoning system of multicellular organisms consists of the entire complement of chaperones, co-chaperones, and chaperone co-factors plus chaperone interactors and receptors, and its components are found throughout the body, intra- and extracellularly, with chaperones having diverse canonical and noncanonical (moonlighting) functions^1–9^. A full array of molecular chaperones is essential in all domains of life to assist the functional folding of their substrate proteins and to protect the cell against the cytotoxic effects of protein misfolding.

It is, therefore, not surprising that chaperone defects can cause human diseases, the chaperonopathies, with complex clinico-pathologic pictures that are, therefore, of interest for physicians across many specialties. Indeed, chaperonopathies, whether genetic or acquired can affect virtually any organ and tissue, showing a variety of signs and symptoms^5,10–17^ (updates at http://www.chaperones-pathology.org, Page BACKGROUND). In many cases, as proven for Hsp70, Hsp90, and the chaperonins (a subset of chaperones), the highly dynamic nature of chaperones and their transient functional interactions to other partners, further complicates interpretation of results^18–22^. So far, the molecular mechanisms that cause the lesions and neuromuscular malfunctions observed in tissues and organs of patients with chaperonopathies involving the chaperonins have not been fully elucidated.

There are at least two lines of experimentation that show the importance of the chaperoning system not only in health but also in disease, and these are the demonstration of distinct interactions between components of the chaperoning system^23^ and the efforts directed toward correcting chaperone defects, for example using chemical compounds^24^.

Although research on the molecular pathogenesis of chaperonopathies is being carried out in a number of laboratories, the field is still at an early stage. For example, for any chaperonopathy, we do not fully understand how a pathogenic mutation, or an aberrant post-translational modification, in a chaperone molecule affects its structure and, thereby, its intrinsic properties and functions. Even partial answers to these questions will contribute to developing therapeutic strategies focusing on the affected chaperone. For instance, if the chaperonopathy is by defect, positive chaperonotherapy would be required, namely the defective chaperone should be replaced (e.g., by gene therapy or administration of purified chaperone) or its weak activity boosted, using chemical compounds for example^24–27^. In contrast, if the pathogenic chaperone contributes actively to the mechanism of disease, negative chaperonotherapy would be indicated and the disease-causing chaperone should be eliminated, downregulated or blocked^24,25,28,29^. In this short review, we will focus on a subgroup of chaperones named chaperonins (Cpn; plural Cpns). They are ubiquitous and build ATP-dependent protein-folding nanomachines with a characteristic double-ring structure; each ring is composed of 7 or 8 subunits of approximately 60 kDa^18,21,22^.

## Chaperonins

Cpns have crucial roles in maintaining protein homeostasis in the cytosol and organelles. The Group I Cpns are conserved in bacteria and mitochondria (e.g., Hsp60 or Cpn60), while Group II Cpns are localized in the cytosol of eukaryotes and cytoplasm of archaea (e.g., eukaryotic CCT or TRiC, and archaeal thermosome, respectively). Recently, Group III Cpns that form a deep, monophyletic branch in the Cpn evolutionary tree were discovered and characterized in Bacteria and some Archaea^30^.

Intensive molecular studies of the Group I Cpn in Bacteria (the GroEL/ES team) have revealed their complex mechanisms of catalysis and allostery in detail^1,7^. The Group II and Group III Cpns^30–32^, which have a related mechanism of articulation, but without a separate lid subunit, are less well understood.

## Chaperonopathies associated with chaperonin genes

The distribution of some of the best known chaperone genes, including those encoding chaperonins, in the three life Domains is illustrated in Table 1. A number of archaeal species do have chaperonins of Group I and all have chaperonins of Group II, therefore, these archaeal microbes are candidates for developing experimental models to study chaperonin functions, mechanisms, and associated chaperonopathies.

**Table 1:** Examples of chaperones, including chaperonins, in the three life Domains

Domain	Chaperone (kDa)						
	≥200	100–199	81–99	65–80	55–64	35–54	≤34
Bacteria	–	–	ClpB	DnaK; HtpG	Hsp60; CCT	DnaJ	sHsp
Archaea	–	–	–	Yes; No^a^	Yes; No	Yes; No	sHsp; Prefoldin
Eukarya	Sacsin	Hsp 100–110	Hsp90	Hsp70; ClpB	Hsp60	Hsp40 (DnaJ)	sHsp; Prefoldin

^a^Some species have it, others do not^59,64–66^. ClpB is present in bacteria and in mitochondria of eukaryotes (see for example https://www.ncbi.nlm.nih.gov/gene/947077 ClpB *Escherichia coli* and https://www.ncbi.nlm.nih.gov/gene/81570 CLPB *Homo sapiens*). HtpG is found in bacteria (see for example https://www.ncbi.nlm.nih.gov/gene/945099 HtpG *Escherichia coli*)

The entire set of chaperonin genes in the human genome has been determined and known chaperonopathies associated with some of those genes are presented in Table 2. The diseases associated with Cpn genetic defects include developmental disorders with profound neurological and muscular abnormalities. Because Cpns are essential components of the protein folding and trafficking machinery, the mutations found in surviving humans cause mild biochemical defects of the chaperonin molecule—it is believed that if a mutation caused a marked structural abnormality it would be lethal, and no phenotype, i.e., patient, would occur. The subtle nature of the viable structural defects create the need for experimental models to study the impact of the pathogenic mutations in chaperonin genes on the subtly altered structure, properties, and functions of the protein products of the mutated genes.

**Table 2:** Diseases associated with mutations in genes encoding chaperonins and in genes phylogenetically related to the CCT family^a^

GENE/Name/HGNC/Gene ID/UniProtKB/Swiss-Prot/Accession number	Chaperonopathies. Mutations
**CCT2**/1615/10576/P78371NM_006431	OMIM:605139. Leber congenital amaurosis (LCA) Mutations T400P and R516H
**CCT4**/1617/10575/P50991/NM_006430	OMIM:*605142. Distal hereditary sensory neuropathy (mutilated foot) in rat. cys450-to-tyr (C450Y)
**CCT5**/1618/22948/P48643/NM_012073.3	OMIM: *610150; #256840. Distal hereditary sensory-motor neuropathy. his147-to-arg (H147R)
**MKKS**/7108/8195/Q9NPJ1/NM_018848.2 and NM_170784.1	OMIM: 04896 gene; 209900 phenotype; 236700 phenotype; McKusick-Kaufman syndrome; MKKS hydrometrocolpos syndrome; hydrometrocolpos, postaxial polydactyly, and congenital heart malformation; HMCS Kaufman-Mckusick syndrome. Y37C (604896.0003), T57A (604896.0010), and C499S (604896.0013): increased MKKS degradation and reduced solubility relative to wild-type MKKS, and the mutant H84Y (604896.0001). R155L, A242S, and G345E mutations: increased MKKS degradation only.
**BBS10**/26291/79738/Q8TAM1/NM_024685.3	OMIM: 610148 gene; 209900 phenotype; Bardet-Biedl syndrome **(r**elationship to Laurence-Moon syndrome). (BBS; 209900) a 1-bp insertion at residue 91 leading to premature termination 4 codons later (C91fsX95). VAL11GLY; ARG34PRO; SER303 FS; SER311ALA.
**BBS12**/26648/166379/Q6ZW61/NM_152618.2	OMIM: 610683 Bardet-Biedl syndrome 12. ALA289PRO; ARG355TER; 3-BP DEL, 335TAG; 2-BP DEL, 1114TT; 2-BP DEL, 1483GA; F372fsX373
**HSPD1**/5261/3329/P10809/NM_199440.1 and NP_955472.1	OMIM: 605280 Spastic Paraplegia 13, autosomal dominant; SPG13, Val98Ile (V98I) Gln461Glu (Q461E)
	OMIM: 612233 Leukodystrophy, Hypomyelinating, 4; HLD4; Mitochondrial HSP60 Chaperonopathy; MitCHAP60 Disease Asp29Gly (D29G)
**HSPE1**/5269/3336/61604.2/NM_002157.2 and NP_002148.1	OMIM: *600141 Neurological and developmental disorder characterized by infantile spasms. Mutation Leu73Phe (L73F)

^a^Reproduced with permission from the ref. ^59^, published under the CC-BY license

## Experimental models for the study of chaperonins

*Saccharomyces cerevisiae* has been used as a model organism for studying a wide range of genetic conditions, even before its genome was sequenced in 1996^33^. It became apparent that many critical biochemical processes in complex Eukaryotes were highly conserved in yeast. For instance, the CCT hexadecamer is formed out of eight dissimilar subunits, which reflect the structure of human CCT. Consequently, the yeast CCT has been used in structural, biochemical, and genetic studies, for example to assess the disparate functions of the eight different paralogs forming the canonical eukaryotic ring^23,34^.

The archaeal Group II chaperonin (from *Methanococcus maripaludis*, *Thermococcus* strain KS-1, and *Pyrococcus horikoshii* OT3) has become a model for allostery, ring opening and closing mechanisms, and for interaction with the co-chaperone prefoldin (GimC)^35–39^; all these mechanistic features are conserved in higher Eukaryotes. However, chaperone defects have not been specifically reproduced in these models yet.

The Hsp60 and Hsp10 that are universally found in mitochondria and in extramitochondrial sites are homologs of bacterial GroEL/ES and have been studied in several human disorders^2,21,24,25,40,41^.

**Fig. 1:**
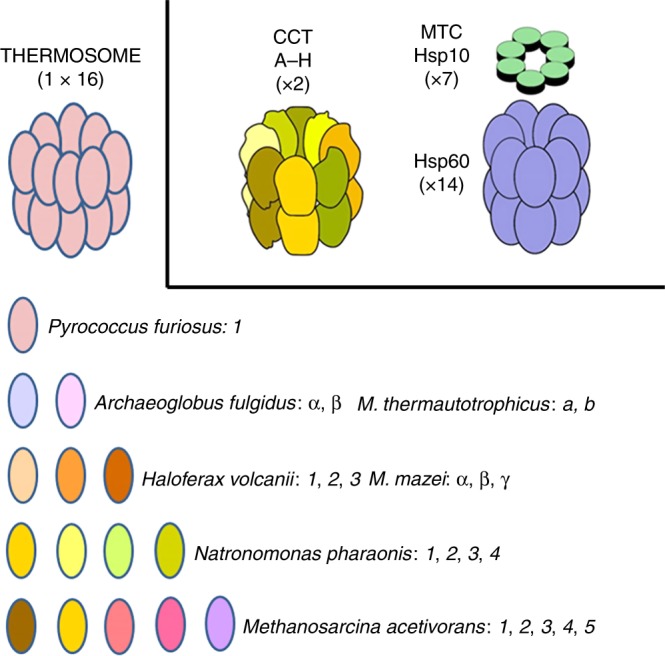
Chaperonins of Group I and II in archaea and humans. Top left: archaeal CCT complex or thermosome; top center the CCT chaperonin of Group II complex typically resident of the eukaryotic-cell cytosol; and top right the chaperonin of Group I (Hsp60 or Cpn60) complex (with Hsp10 on top) characteristic of bacteria and eukaryotic-cell mitochondria. Archaeal species vary in their content of chaperonin genes–proteins from only one through a maximum (at least from what we know at the present time) of five. These subunits are variously designated with Arabic numbers, English letters, or Greek letters. As far as we know, they all form a hexadecameric megadalton sized complex, the thermosome, of the type shown on top to the left, which is an example of a hexadecamer found in archaea encoding only one chaperonin subunit, e.g., *P. furiosus*. The composition of hexadecamers in all archaea that have two or more subunits is not yet fully elucidated. *M. thermautotrophicus*, *Methanothermobacter thermautotrophicus* Δ*H*, previously known as *Methanobacterium thermoautotrophicum* Δ*H*. *M. mazei*, *Methanosarcina mazei*. (Reproduced with permission from ref. ^59^, published under the CC-BY license)

Recently, thermophilic microbes have been used to study pathogenic, heritable mutations in human chaperonins. For example, in CCT5 (Table 2) the single amino acid change, His147Arg, causes a serious peripheral sensory neuropathy^42,43^ and has been characterized using recombinant human CCT5 subunits^44,45^. We modeled the mutation in the hyperthermophilic Archaeon, *Pyrococcus furiosus* Cpn (Pf-Cpn), and examined its molecular effects^46,47^. *P. furiosus* has only one Cpn gene, Fig. 1, which is a CCT subunit ortholog with conservation of the hexadecameric complex and the mutation site, thus the effect of the mutation was amplified eightfold in the homogeneous archaeal complex (octameric ring), enabling the mechanism of action to be examined^46,47^.

Another experimental model recently developed uses the thermophilic fungus *Chaetomium thermophilum*: the entire CCT complex from this microbe has recently been expressed in recombinant form, which allowed the study of ring closure at single-molecule scale^48,49^. Proteins from the thermophilic eukaryote *C. thermophilum*, like those from thermophilic archaea such as *P. furiosus*, exhibit structural stability compared with those from mesophilic organisms and are, therefore, convenient to study the effect of mutations. Because they have an extraordinary margin of reserve stability, the deficits of stability caused by pathogenic mutations are tolerated, enabling correct assembly of the hexadecamer. The thermophilic fungus, *C. thermophilum* thrives optimally at 50–55 °C, and its proteins are highly stable, as shown experimentally. It is pertinent to emphasize that the thermostability of the molecules from *C. thermophilum*, including its chaperones, facilitates manipulation of single molecules and insights into functional multimolecular complexes.

## Group III chaperonins in bacteria

One of the most interesting aspects of the evolution of the chaperonins is the existence of two sequence-related Cpn families with strong evidence for descent from a common ancestor, yet the ancestral forms are not accessible. A new deep-branching clade of Cpns was identified first encoded in bacteria^30^ and the prototype Cpn has recently been crystallized and the mechanism of ring opening and closure has been observed by members of our group^31,32^. This Cpn is phylogenetically ancient and lacks some key allosteric mechanisms identified in the Group II Cpn. It is arguably a remnant of an early branch of the Group II Cpn clade and, thus, it provides insights into the evolution of the Cpn within the Archaea that predated extensive gene expansion resulting in the canonical eukaryotic CCT, which is formed with eight paralogs in each ring of the hexadecamer. The mechanism of ring closure and opening was analyzed by obtaining high-resolution crystal structures of the open forms (soaked in ADP) and closed forms (soaked in the nonhydrolyzable ATP analog AMP-PNP). The opening function, which is a highly cooperative and orchestrated allosteric cascade in other Cpn60 paralogs that have been analyzed to date, was instead essentially noncooperative in the Group III chaperonins. This provides another opportunity to examine the evolutionary progression of the cooperative kinetics observed in the modern homologs of Cpn60 that have been studied, for example, by high-resolution crystal structures and analysis of client protein specificity^50–52^. The bacterial version of the Cpn has the advantage that it has evolved in the absence of prefoldin (Pfd) and, therefore, effects of co-chaperone interaction will presumably be absent in its mechanism.

## The future potential of Methanosarcinales and Sulfolobales

Genes encoding chaperonins of Group II are present in all archaeal species whereas those encoding chaperonins of Group I occur only in some species. Archaea with both Groups of chaperonins are listed in Table 3. The number of Cpn subunit genes (CCT orthologs) in archaeal genomes varies from 1 to 5, but the latter high number has been found only in one archaeal species, *Methanosarcina acetivorans*^53,54^.

**Table 3:** Examples of archaea with both, Group I and II chaperonin genes^a^

Organism	Chaperonin genes of Group	
	I	II (subunits)
*Methanococcus vannielii* SB	1	1
*Methanospirillum hungatei* JF-1	1	2
*Methanosarcina barkeri* str. Fusaro	1	3
*Methanosarcina mazei* Go1	1	3
*Methanosarcina acetivorans* C2A	1	5

^a^Reproduced with permission from ref. ^59^, published under the CC-BY license

Regardless of the number of CCT subunits in the archaeal genomes, the archaeal CCT complex, also referred to as the thermosome, has in all the species studied thus far the same overall structure as that of the eukaryotic equivalent: two octameric rings stacked together to build an hexadecamer, Fig. 1. *M. acetivorans* is a potentially convenient experimental model to examine, for instance, hexadecameric thermosomes formed by only one of its five subunits or by various combinations between them to elucidate if they have distinct substrate spectra^54^. In this regard, it is of interest that the individual *M. acetivorans* (METAC) thermosome subunits phylogenetically cluster preferentially with one or the other of the human (Hs) orthologs as follows: METAC subunits 4 and 5 cluster together and with HsCCT6A and HsCCT6B; METAC3 with HsCCT8; and METAC1 and METAC2 are more distant from the human orthologs and cluster with other archaeal thermosome subunits. This suggests that some *M. acetivorans* subunits would be preferable with regard to the others as experimental models to study the impact of pathogenic mutations or post-translational modifications on any single human CCT subunit, depending on which human subunit is mutated or modified in disease.

Specialization of hexadecamers has been observed in some species of the hyperthermophilic archaeal genus Sulfolobales that have three Cpn subunits^55^. Three different complexes were found with preference for different sets of substrates depending on growth temperature. The α and β subunits form a generalized heterocomplex with a high limit for thermostability, whereas the γ subunit forms an exclusive 16-mer complex with a Tm for denaturation lower than the growth optimum of the organism (85 °C). This is a special case since *Sulfolobus* spp. are extremely acidophilic hyperthermophiles with an exceptionally wide ranges of growth temperature and pH tolerance and may have evolved the set of CCT complexes to accommodate for both temperature and acid stress conditions.

Other archaeal species with more than one Cpn (CCT) subunit, such as *M. acetivorans*, *M. mazeii*, and others (Table 3, and Fig. 1), are convenient for studying various combinations of subunits, while species with only one thermosome subunit, such as *P. furiosus*, are convenient to amplify the effect of a mutation in the single subunit on the octameric ring assembly and on the entire hexadecamer.

## Specific examples of models for eukaryotic heaxadecamers

### Studies with *P. furiosus*

**Fig. 2:**
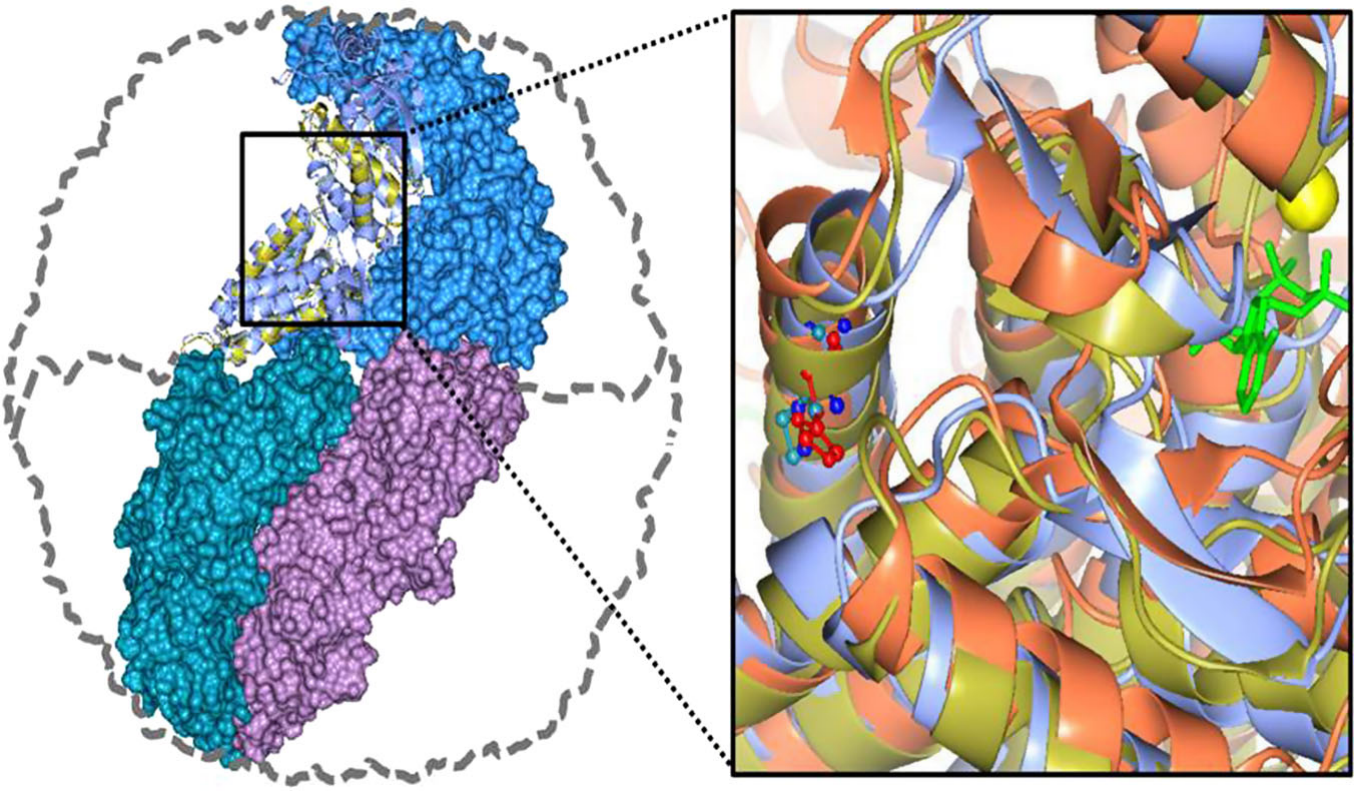
Human CCT5 and *P. furiosus* Pf-Cpn (Pf-CD1) superposed onto the crystal structure of *Thermococcus* strain KS-1 subunit α (1Q3Q). Left: The Pf-CD1 graphic representation (gold ribbon) was obtained in Swiss-Model (http://swissmodel.expasy.org/) and superposed onto the crystal structure of KS-1 subunit α (monomers are displayed in marine blue, violet, and deep teal colors as surface, and in cyan color as ribbon). The whole hexadecamer double-ring structure is depicted by a dotted line. Right: Magnified image of the superposed structures of the Pf-CD1 (gold ribbon) and Human CCT5 (orange ribbon) onto the KS-1 α subunit crystal structure (1Q3Q; cyan ribbon). Side chains of isoleucine at 138 of Pf-CD1 (blue), isoleucine at 138 of KS-1 subunit α (deep teal), and histidine at 147 of human CCT5 (red) are represented as ball and stick. AMP-PNP (β,γ-Imidoadenosine 5′-triphosphate lithium salt hydrate; green stick) and magnesium ion (yellow ball). (Reproduced with permission from ref. ^46^, published under the CC-BY license)

**Fig. 3:**
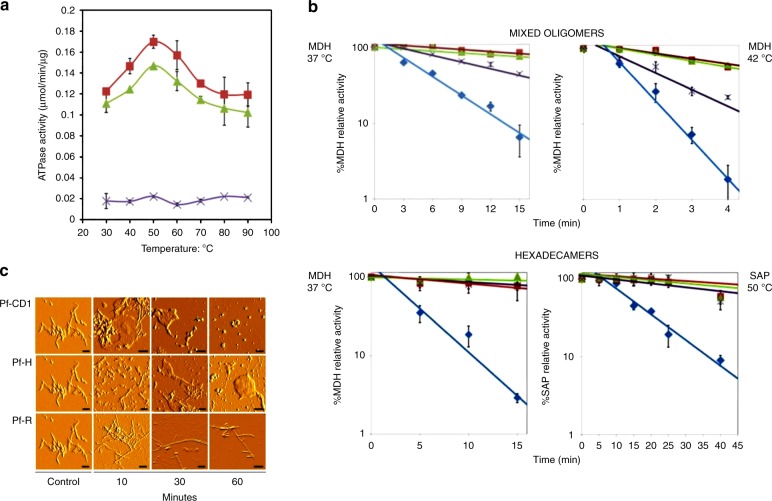
**a** The pathogenic mutant causes a loss of ATPase activity evidenced at various temperatures. Pf-CD1 (red squares), Pf-H (green triangles), and Pf-R (purple stars). The results shown are mean values (±standard deviations) and the experiments were carried out in triplicate. See ref. ^46^. **b** Comparative analyses of protective capacity of mutant and wild type chaperonins. Protection from heat denaturation of malate dehydrogenase (MDH) by mixed oligomers of Pf-CD1 (red square), Pf-H (green triangle), and Pf-R (purple star) at 37 °C (top left panel) and 42 °C (top right panel); and protection of MDH at 37 °C (bottom left panel) and shrimp alkaline phosphatase (SAP) at 50 °C (bottom right panel) by pure hexadecamers. Negative control (no chaperonin added), i.e., MDH, or SAP in bottom right panel, alone: blue diamond. The results shown are mean values (±SD) of triplicate experiments. See ref. ^46^. **c** The pathogenic mutant fails to disperse amyloid fibrils. Dispersion of amyloid fibrils by archaeal Pf-CD1 (top row of panels), partial dispersion by Pf-H (middle row of panels), and no dispersion by Pf-R (bottom row of panels). Atomic force microscopy (AFM) of bovine insulin amyloid fibrils treated with Cpn and Mg++, and ATP. Control panels, no added chaperonin. Scale bar: 250 nm. (Reproduced with permission from ref. ^46^, published under the CC-BY license)

As shown in Fig. 1, the thermosome of *P. furiosus* is a hexadecamer formed by the only CCT subunit ortholog (Pf-Cpn) of this organism. We have used this model to investigate the effects of the pathogenic mutation His147Arg in the human CCT5 subunit on the properties and functions of the Pf-Cpn and the complexes (oligomers) it forms^46,47^. First, we determined the position in the Pf-Cpn that matches His145 in the human CCT5. We found it is Ile138, as illustrated in Fig. 2. Then we made a mutant Pf-Cpn in which we introduced the neutral mutation Ile138His (Pf-H) and another in which the mutation was Ile138Arg (Pf-R). In the latter, which is equivalent to the His147Arg pathogenic mutation, the ATPase activity was drastically reduced, Fig. 3a. We also measured the potential of this mutant for protecting other proteins from heat-induced denaturation and illustrative results are shown in Fig. 3b. It can be seen that protection by the mutant Pf-R was deficient when mixed oligomers were used, while it was the same as that of the wild type when purified mutant hexadecamers were tested. This, along with data from gel electrophoresis, indicated that the mutation affected the capacity of the Pf-R subunit to oligomerize and associate into functional hexadecamers but, if the latter were formed, they were as efficient as wild type counterparts. Also, mixed oligomeric preparations of Pf-R were deficient in dispersing insoluble amyloid fibrils, Fig. 3c. A summary of results is displayed in Table 4.

**Table 4:** Summary of results showing the functional performance of the pathogenic mutant Pf-R in comparison with nonpathogenic counterparts^46^

Gene	*E. coli* exp.	Mixed oligomers						Hexadecamers	
		Heat res.	ATPase	Disperse fibrils	Form hexadecamers	Protection			
						MDH		MDH	SAP
						37 °C	42 °C	37 °C	50 °C
Pf-CD1	100	100	100	100	100	100	100	100	100
Pf-H	100	100	100 or less	100 or less	100 or less	100	100	100	100
Pf-R	100	100	Low	Low	Low	Low	Low	100	100

*exp.* expression, *res.* resistance, *100* optimal, *MDH* malate dehydrogenase, *SAP* shrimp alkaline phosphatase

In order to quantify the impact of the pathogenic mutation in CCT5, quantitative biophysical analysis on the archaeal model chaperonin was carried out using differential scanning microcalorimetry, isothermal titration calorimetry, and gel permeation chromatography^47^. The results showed the following: ‬The archaeal ‬chaperonin was found to be a good fit to a theoretical model based on ‬16 ‬identical ‬nucleotide ‬binding ‬sites, ‬which ‬were ‬considered ‬to ‬be independent ‬of ‬each ‬other ‬with ‬a ‬uniform ‬binding ‬constant, K‬a. ‬The ‬theoretical ‬curves ‬showed ‬a ‬good ‬match ‬with ‬the ‬experi‬‬‬‬‬‬‬‬‬‬‬‬‬‬‬‬‬‬‬‬‬‬mental ‬data ‬for ‬binding ATP during isothermal titration calorimetry. ‬Interestingly, the ‬three ‬proteins ‬showed ‬different ‬ATP-binding mechanisms, in which ‬Pf-R, the model for the human pathogenic mutation, ‬showed ‬an ‬exothermic ‬binding ‬enthalpy ΔH (−‬‬26.8 kcal/mol) ‬compared ‬to ‬the ‬endothermic ‬ones ‬of ‬Pf-CD ‬and‬‬ Pf-H ‬that ‬were ‬4.3 ‬and ‬6.5 ‬kcal/mol, ‬respectively.‬‬‬‬‬‬‬‬‬‬‬‬‬‬‬‬‬‬‬‬‬‬‬‬‬‬‬‬‬‬‬‬‬‬‬‬‬‬‬‬‬‬‬‬‬‬‬‬‬‬‬‬‬‬‬‬‬‬‬‬‬‬‬‬‬‬‬‬‬‬‬‬‬‬‬‬‬‬‬‬‬‬‬‬‬‬‬‬‬‬‬‬‬‬‬‬‬‬‬‬‬‬‬‬‬‬‬‬‬‬‬‬‬‬‬‬‬‬‬‬‬‬‬‬‬‬‬‬‬‬‬‬‬‬‬‬‬‬‬‬‬‬‬‬‬‬‬‬‬‬‬‬‬‬‬‬‬‬‬‬‬‬‬‬‬‬‬‬‬‬‬‬‬‬‬‬‬‬‬‬‬‬‬‬‬‬‬‬‬‬‬‬‬‬‬‬‬‬‬‬‬‬‬‬‬‬‬‬‬‬‬‬‬‬‬‬‬‬‬‬‬‬‬‬

Our ‬results ‬indicate ‬that ‬the mutation introduced a defect that restricted the‬‬‬‬ conformational ‬changes ‬induced ‬by ‬Mg-ATP during the progression to ‬the ‬closed ‬conformation. ‬Circular dichroism ‬results ‬also showed ‬that ‬the ‬three ‬chaperonin ‬variants ‬undergo ‬distinct conformational ‬changes ‬upon ‬ATP ‬binding. ‬Pf-CD1 ‬and ‬Pf-H ‬became ‬more structured ‬upon ‬ATP ‬binding, ‬whereas ‬Pf-R ‬lost ‬structure ‬and ‬resembled ‬the ‬denatured ‬state. ‬The ‬pathogenic ‬R ‬mutant ‬(Pf-R) ‬was ‬energetically ‬the ‬weakest ‬at ‬maintaining ‬the ‬oligomeric ‬complex, confirming that its functionality was compromised by the presence of Arg in the mutation site.‬‬‬‬‬‬‬‬‬‬‬‬‬‬‬‬‬‬‬‬‬‬‬‬‬‬‬‬‬‬‬‬‬‬‬‬‬‬‬‬‬‬‬‬‬‬‬‬‬‬‬‬‬‬‬‬‬‬‬‬‬‬‬‬‬‬‬‬‬‬‬‬‬‬‬‬‬‬‬‬‬‬‬‬‬‬‬‬‬‬‬‬‬‬‬‬‬‬‬‬‬‬‬‬‬‬‬‬‬‬‬‬‬‬‬‬‬‬‬‬‬‬‬‬‬‬‬‬‬‬‬‬‬‬‬‬‬‬‬‬‬‬‬‬‬‬‬‬‬‬‬‬‬‬‬‬‬‬‬‬‬‬‬‬‬‬‬‬‬‬‬‬‬‬‬‬‬‬‬‬‬‬‬‬‬‬‬‬‬‬‬‬‬‬‬‬‬‬‬‬‬

### Studies with *C. thermophilum*

**Fig. 4:**
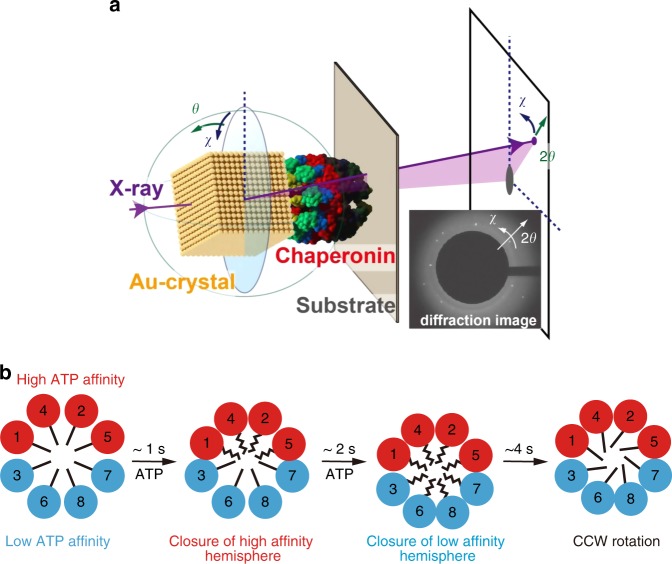
Schematic representation of the asymmetric conformational changes in the CCT revealed by diffracted X-ray tracking (DXT) and using the *C. thermophilum* model. **a** Internal motions of group II chaperonins by DXT^63^. **b** Asymmetric conformational changes in the CCT. Subunits are colored according to nucleotide affinity as follows: red, high; blue, low. The conformational change proceeds in a sequential manner owing to the asymmetric consumption of ATP by the eukaryotic group II chaperonin. Firstly, ATP binds to the high ATP affinity hemisphere and induces the conformational change, in less than one second. Subsequently, the low-ATP affinity hemisphere changes to the closed conformation. Finally, the ring rotates in counter clockwise direction. (Reproduced with permission from ref. ^48^, published under the CC-BY license)

So far, recombinant human CCT has been obtained by expression in *S. cerevisiae* or by expressing an individual subunit containing a specifically tagged human CCT subunit in a hamster BHK-21 cell line^56,57^. Generally, human proteins are relatively unstable and these recombinant versions are no exception, especially, the pathogenic mutant CCT proteins that are difficult to express and characterize because of their structural instability. The expression of CCT subunits from *C. thermophilum* in *Escherichia coli* and the assembly of the hexadecamer have been achieved^48^. Taking advantage of its structural stability, the functional mechanism of the *C. thermophilum* CCT was analyzed by various biophysical techniques^39,58^. Mutant CCT complexes containing individual specific subunits with amino acid changes were constructed and analyzed. The ATP-dependent motion of CCT from *C. thermophilum* was examined at the single-molecule level by diffracted X-ray tracking (Fig. 4a). The results supported the notion that ATP-dependent conformational change starts with the high-affinity hemisphere and progresses to the low-affinity hemisphere (Fig. 4b). The system will be useful for analyzing the effects of pathogenic mutations on the conformational progression of the wave of ATP hydrolysis around the octameric rings of the Cpn complex.

The interaction between CCT and its substrates actin or tubulin is different from that of bacterial and archaeal chaperonins with their substrates and occurs via specific CCT subunits, suggesting that each subunit has a specialized role^56–60^. CCT is known to be involved in the folding of 5–10% of newly synthesized cytosolic proteins. Therefore, it is reasonable to think that pathogenic mutations will impair the folding of a specific client protein. To study the effect of pathogenic mutations in CCT, construction of recombinant CCT containing a mutant subunit is necessary.

The Group II chaperonin cooperates with prefoldin as described for human CCT and prefoldin^57,58^. As a working hypothesis we consider that at least some CCT-related chaperonopathies might be due to the impairment of the CCT cooperation with prefoldin. Eukaryotic prefoldin is a multisubunit chaperone, with a hexameric complex assembled from six different subunits^61,62^. Recently, a hexameric prefoldin complex from *C. thermophilum* (CtPFD) was successfully reconstituted^49^. The interaction between CtPFD and CCT from *C. thermophilum* (CtCCT) was analyzed by surface plasmon resonance and the *K*_D_ value is similar to that of the archaeal counterparts^49^. The system will be used to analyze the effects of pathogenic mutations of CCT/PFD folding pathways affecting the maturation of specific client proteins such as actin and tubulin.

## Limitations

The experimental models discussed involving *P. furiosus* and *C. thermophilum* are convenient in various ways, as noted in the preceding sections, but they have some limitations at this time in their development. These pertain to the limited availability of genetic systems and the unusual requirements for their culture in vitro. However, both issues are under intense research and the future is promising as indicated by several genetic systems already standardized and the familiarity of an increasing number of laboratories with the techniques for growing archaea and *C. thermophilum*; many of these laboratories have limitations and concerns regarding the use of human materials and, therefore, prefer to work with microbial models such as the ones described here. Consequently, standardization of the techniques involved is a current priority: for instance calibration of growth conditions of archaea and *C. thermophilum*, optimization of culture media suitable to the purpose of the experiments, and purification and characterization of target proteins. These technical developments are some of the main objectives in research on chaperonopathies along with the elucidation of the impact of mutations and post-translational modifications on the structure, properties, and functions of the pathogenic chaperones.

## Conclusions and perspective for the future

Microbial chaperones, including Cpns from archaea and the fungus *C. thermophilum*, can be useful in elucidating key aspects of chaperonopathies as a way to developing biochemical tools for accurate characterization of molecular kinetic and stability defects, for clinical diagnosis and assessing prognosis and response to treatment, and for standardizing therapeutic means centered on chaperones. The microbial chaperonins discussed here and human chaperonopathies are not as distant from one another as may appear at first: they can be efficiently bridged by research aimed at gaining understanding of the latter and developing means for their treatment. For instance, data obtained with the *P. furiosus* model showed that a pathogenic mutation in the CCT5 subunit weakens its ability to form the typically functional hexadecamers and, thereby, impairs its chaperoning ability. Likewise, other deficits of the mutant chaperonin molecule were brought to light by the model, such as attenuation of its ATP hydrolysis potency, likely connected to ‬an ‬exothermic ATP ‬binding ‬enthalpy that contrasted with the endothermic enthalpy of the wild-type molecule.

Another important property of CCT subunits is formation of functional multichaperone complexes and networks, for instance with prefoldin. We have shown that chaperonin–prefoldin interactions can be investigated using *C. thermophilum*, which can also be done with archaea that have prefoldin. It is likely that mutants have an impaired ability for networking with other chaperones, much as individual subunits have a decreased capacity to interact with one another and build efficient chaperoning complexes (e.g., hexadecamers in the case of CCT).

Future research ought to continue on the clarification of the impact of mutations and aberrant post-translational modifications on the pathogenic chaperonin molecules, including the standardization of experimental models amenable to molecular and genetic manipulations in vitro as well as in vivo. The latter, in vivo research will be instrumental to elucidate the molecular underpinnings of the tissue and organ lesions observed in patients with chaperonopathies. The experimental models described here in some detail (and similar ones involving the other archaea mentioned earlier, e.g., Methanosarcinales) will enable future researchers to dissect the specific biochemical and cellular effects of the defective, malfunctional chaperonins causing disease. Elucidation, at the molecular level, of the tissue pathogenic steps in which defective chaperonins are involved will provide information essential for designing and testing treatment strategies and chemicals centered on the chaperonins and, for this purpose, microbial experimental models will be instrumental.
